# Barriers to gene therapy, understanding the concerns people with haemophilia have: an exigency sub-study

**DOI:** 10.1186/s13023-024-03068-2

**Published:** 2024-02-10

**Authors:** Simon Fletcher, Kathryn Jenner, Michael Holland, Kate Khair

**Affiliations:** 1 Haemnet, London, N15 3JR UK; 2 Oxford Haemophilia and Thrombosis Centre, Oxford University Hospitals NHS Foundation Trust, Oxford, OX3 7LE UK

**Keywords:** Haemophilia, Gene therapy, Shared decision-making, Outcomes, Quality of life

## Abstract

**Background:**

Gene therapy has the potential to offer people with haemophilia (PwH) a life free from bleeding and the burden posed by current treatment regimens. To date, gene therapy has only been available in clinical trial settings, to PwH without pre-existing or historical factor inhibitors, significant concomitant liver damage or pre-existing neutralising antibodies to the adeno-associated viruses used to deliver the therapy. Thus, most PwH treated at centres not currently involved in gene therapy trials, either as a referral/follow-up centre or as a dosing centre, have been unable to access the therapy. This Exigency sub-study aims to gain a greater understanding of the opinions of PwH in the United Kingdom who have not had access to gene therapy: asking what they understand, what concerns they have, and whether they perceive any barriers preventing their access to gene therapy.

**Results:**

Twenty-three PwH were approached; 14 consented, and one withdrew prior to interview. The mean age of the participants was 35.7 years (range 25–74 years). Eleven had haemophilia A and two haemophilia B. Two were treated with standard half-life factor products, five with extended half-life products, five with a FVIII mimetic and one with a clinical trial product. One family member (a participant's partner) was also interviewed. The participants identified four barriers to gene therapy: concerns about the process of gene therapy (Expectations), uncertainty about the results (outcomes), (Access) to treatment, and a lack of understanding about gene therapy (education).

**Conclusions:**

This Exigency study subgroup sees gene therapy as a positive treatment development that promises an improved quality of life. For this participant group, four issues impact their decision to undergo gene therapy. If the promise of gene therapy is to be realised, these barriers need to be acknowledged and addressed by healthcare professionals, patient organisations, and gene therapy providers.

**Supplementary Information:**

The online version contains supplementary material available at 10.1186/s13023-024-03068-2.

## Background

Gene therapy offers people with haemophilia (PwH) the potential of a life free from prophylactic factor replacement therapy and spontaneous joint bleeding [1, 2]. The availability of gene therapy for haemophilia has, however, been constrained as it has only been available as part of a clinical trials programme. Restrictive inclusion and exclusion criteria including age (≥ 18 years), pre-existing or historical inhibitors, significant concomitant liver damage and pre-existing neutralising antibodies to the adeno-associated vector (AAV) used have also limited its availability [1, 3–5].

Two gene therapy products have now been granted marketing approval in Europe and the United States [6, 7] with more expected in the next 12 months [8]. While it is likely that many of the restrictive inclusion and exclusion criteria seen in the clinical trials will be maintained in the marketed product, a growing number of PwH will soon be able to access gene therapy. The hub and spoke model of care proposed by the European Haemophilia Consortium and the European Association of Haemophilia and Allied Disorders [9, 10], will mean that those centres not previously part of a trials programme will be able to access the specialist expertise necessary to facilitate access to gene therapy for the PwH in their care.

Qualitative studies have explored the impact of gene therapy on those who have had it [11–13] and those who were excluded from having it [14]. A number of studies have also examined what the wider haemophilia community thinks of gene therapy and whether it is a treatment they would consider [15, 16]. This sub-study, part of the larger Exigency study [17], seeks to build on this body of knowledge by investigating perceptions of and concerns about gene therapy among PwH in the United Kingdom (UK) who have not yet been able to access it, and to identify the barriers to access they perceive if and when it becomes a standard of care (SoC) treatment option.

## Results

We approached 23 PwH and consented 14. One withdrew before interview. Recruitment was discontinued after 13 interviews (participant codes Exi201–Exi213) as we deemed data saturation had been achieved. The method described by Guest, Namey and Chen, was used to assess saturation [18]. Five data collection events were used to calculate the base size (105), with three data collection events per run length of two interviews. A < 5% new information threshold was used for the level of saturation confidence (see Table 1).

**Table 1:** Data saturation calculation

Interview Number	1	2	3	4	5	6	7	8	9	10	11	12	13
New themes per interview	22	19	23	18	23	14	12	9	8	5	2	3	1
# Base themes					105								
New themes per run						26		17		7		4	
% New terms						24.8%		16.2%		6.7%		3.8%	

The mean age of participants was 35.7 years (range 25–74 years). Eleven had haemophilia A; two had haemophilia B. All participants were on prophylaxis, two on standard half-life (SHL) factor products, five on extended half-life (EHL) products, four on a FVIII mimetic and one on a clinical trial product. Demographic and treatment details for the participants are given in Table 2**.**

**Table 2:** Biographical and treatment data

	Exi201	Exi202	Exi203	Exi204	Exi205	Exi206	Exi207	Exi208	Exi209	Exi210	Exi211	Exi212	Exi213
Age	74	32	57	42	23	29	26	25	25	53	24	24	30
Haemophilia Type	A	A	A	A	B	A	A	A	A	A	A	A	B
Ethnicity	White	White	White	Asian	White	White	White	White	White	White	White	White	White
Treatment Centre	HTC	CCC	CCC	CCC	CCC	CCC	HTC	HTC	HTC	CCC	HTC	CCC	CCC
Current treatment	Extended Half-Life Product	FVIII Mimetic	FVIII Mimetic	FVIII Mimetic	Extended Half-Life Product	FVIII Mimetic	Extended Half-Life Product	Standard Half-life Product	Extended Half-Life Product	Standard Half-life Product	Extended Half-Life Product	FVIII Mimetic	Clinical Trial Product

*HTC* haemophilia treatment centre, *CCC* comprehensive care centre

All participants saw gene therapy as a positive development and one that had the potential to improve the quality of life (QoL) of PwH,

'I think its advancement in treatment. It's the next stage in treatment.'

[Exi204]

There's people who'd benefit from that [gene therapy] to give them a better quality of life.

[Exi207]

There were, however, four key barriers which they suggested might prevent them and others from having gene therapy:

- *Expectations*: concerns about the process of gene therapy
- *Outcomes*: uncertainty about the results
- *Access* to treatment
- *Information*: a lack of information about gene therapy.

### Expectations

Despite having a limited understanding of all the processes involved in gene therapy, many participants were aware of some and had concerns about them. Ten were worried about possible side effects and associated consequences of having gene therapy. Three participants knew of people who had had gene therapy and required steroids to maintain factor expression. Their concerns centred on the side effects they had seen, including weight gain, insomnia and immunosuppression, a particular concern during the Covid-19 pandemic.

'I did some reading and then I also know just from social media what it was like to go through the process, and I just thought the process looked awful, really, to be honest.'

[Exi205]

'And granted, the circumstances of [the individual] but also when he went on it he had to go on immunosuppressants, which at the time of Covid and all that stuff it… Covid in itself was just another big issue.'

[Exi212]

Three participants were concerned about the level of commitment and engagement that gene therapy would involve. Two acknowledged that it had the potential to improve their overall QoL but believed it would also increase their treatment burden, particularly in the short term.

'The fact is I really enjoy the treatment I'm on. I chose this treatment because I can't be bothered dealing with haemophilia – I want to just treat and forget. And obviously, that's maybe one of the selling points of gene therapy, but not in its current formation when there's so much baggage attached to it.'

[Exi202]

'I just can't be bothered with the faff [inconvenience], to be honest.'

[Exi211]

Three participants thought that the commitment required for follow-up was manageable on a personal level, but two did not think their employers would be as accommodating, potentially impacting their ability to have gene therapy.

'For me, it would be fine. Whether my employer had the same sort of mindset.'

[Exi208]

### Outcomes

Five participants expressed concerns about the outcomes of gene therapy, with two citing rumours that the factor levels achieved were not as good as they would wish:

'To be fair, if someone said to me, "Ok, we'll go for gene therapy, your levels are going to be 60," I'd jump at it now. But the fact of, "Ok, we don't know what's going to happen and how you're going to react to it," it's too much of a risk for me at this moment in time.'

[Exi206]

Others had heard that the treatment was not as durable as they would hope. One reported he would sooner *'wait until there was more longevity data available’* [Exi208] before being ready to decide to have gene therapy.

Four participants stated that gene therapy would impact their independence and lead to an increased reliance on their treatment centre:

'I knew exactly how to look after myself, for lack of a better term. It wasn't the best treatment for me because I was treating daily. However, I knew exactly how to play it, and I knew if I did my injection in the morning I was covered. When I moved to [FVIII mimetic], I went from treating every day to once a week and then to fortnightly, and I'll go away for a weekend or I'll go and do something and I get paranoid that I'm not covered because I'm so used to having that injection and knowing that I've topped my levels up and I can go and do this thing. So, I think taking gene therapy and taking the injections entirely out of the equation might be a little bit too much.'

[Exi206]

'Right now, I think, my life is quite settled in the sense of I'm treating every fortnight, I'm doing it on my own, I don't need to go to my treatment centre other than for my six-month check-up.'

[Exi212]

Three participants were concerned that rather than reduce their treatment burden, gene therapy would add an administrative burden to their lives they were unwilling to accept:

‘The biggest impact haemophilia has on me isn't the bleeding, it's not even the joint damage, it's all the paperwork, it's all the bureaucracy, the admin, managing hospital appointments, managing home deliveries that never quite go right. So, any more paperwork, like haemophilia admin […] I don't see the need to take on any more bureaucracy. I don't see what gene therapy is going to add to pay that value, pay that extra cost.'

[Exi202]

‘[There would be] a lack of independence because I’d have to ring my centre – “I’ve got a bleed. What do I do?” where I’ve spent so long being like, “I’ll do what I want. I’m going to do something stupid, so I’ll take an extra dose.”’

[Exi206]

'I think there's going to be so much health surveillance attached to it right now, compared to I do a subcut injection twice a month and have a five-minute clinic conversation once every six months. Why would I trade that in?'

[Exi207]

One participant even stated the treatment he received, as someone with severe haemophilia, was better than he would have after gene therapy as a person with mild haemophilia.

'I'm very much of the belief that because treatment's available to severe haemophiliacs I sometimes have a better quality of life than some with mild and moderate [Haemophilia]. So, I wouldn't want to go through all that to then become essentially a mild haemophiliac, if you will, when mine is much more manageable because the treatment is there.'

[Exi206]

### Access

Eleven participants had concerns about who would decide which PwH would be offered gene therapy and on what grounds any decision would be made.

None of the participants in this sub-study were treated at centres involved in gene therapy clinical trials and they had, therefore, been unable to access gene therapy. Two acknowledged that their centre was not large enough to have been involved in gene therapy studies; however, three believed their centre was large enough to have been involved but had actively decided not to participate in the gene therapy clinical trials. They also thought their centre would be unlikely to offer gene therapy if and when it becomes available as a SoC treatment option.

'I think [my centre] has made a decision that "We're going to get all our patients we can onto [names product]".'

[Exi204]

'It's almost like you are steered towards a particular treatment or a particular brand of treatment.'

[Exi203]

Three participants stated that their care team made treatment decisions in good faith, based on benefit to the individual; for example, because they *'last longer in your system, you're getting fewer bleeds now’* [Exi203]. Three, however, said this was not always the case, and two said that despite having compelling reasons to switch treatment, their care teams were not always willing or able to consider it.

'The treatment that I am on, I had to argue heavily to get onto it. I'd spent a few years asking for an extended half-life, was getting told, "No, your current treatment's working for you." And it was working for me because I was fiddling the doses I was taking, and I told them that.'

[Exi206]

One described having never been aware that a choice was available, saying it had always been a case of,

'"this [treatment] is coming, we're about to sign a contract, this is the plan." So, it's more about, "This is the plan, this is what we're going to do, and at some point you'll move across to this."'

[Exi204]

Five were aware that, ultimately, cost drives treatment choice and availability of treatment.

'I know the NHS is always focused on this cost-price analysis about…you know, the utilitarian argument about how do we get the best gain with the smallest amount of money.'

[Exi205]

As a result, they felt that, in reality, they had little say over what treatments they could access. Most did not think the advent of gene therapy would change this due to its cost.

'Unfortunately, I personally believe it's always going to come down to cost as number one. And I know that might make me come across a bit bitter, but I think it will always be cost one, patient two.'

[Exi208]

'From everything I've heard about gene therapy, it costs a lot of money.

[Exi205]

Two participants thought the cost of gene therapy would be so prohibitive that it would be better to direct research into treatments that have the greatest impact on the greatest number of people with haemophilia.

### Information

Though all the participants had been aware of gene therapy for more than a decade, seven said they were concerned about the lack of easily accessible and understandable information. Although four had searched for information about gene therapy online, one said the propensity for '*misinformation on the internet*' [Emi210] made him reluctant to use it as a resource. Four participants stated that even when information was available, much of it was too complicated.

'I think if there'd have been more communication, different communication, more layman's term communication, that would have enabled haemophiliacs to make a more informed choice on their treatment, that would have been great.'

[Exi203]

'The language [is] still very medically orientated and not for the layman.'

[Exi207]

One participant said that some healthcare professionals (HCPs) were bad at explaining the therapy’s complexities in an easily digestible form.

'I ask a question…a 30-second question and get a 25-minute answer. [my consultant] can be half an hour, 40 minutes, 50 minutes, and by that time… What I would love is simple stuff. Simple, just easy for me to break down, easy for me to understand, easy for me to make a decision on.'

[Exi203]

Two participants thought there was a lack of information simply because it was not known.

'I think they should be putting more information out, but I'd like to know if they actually have the information themselves. That's the thing I'm really curious about because I'm not so sure they do. I'm not sure they've potentially got this actual data to show us all yet.'

[Exi205]

Three participants said that clinicians at their centres did not have the time to provide information about gene therapy, meaning they lacked the knowledge needed to make any decision about gene therapy as a treatment option.

'I only have a five-minute conversation with my consultant anyway, about every six months. He phones me up and says, "Are you doing all right?" and I say yes. He says, "Have you had any bleeds?" and I say no. And that's it.'

[Exi201]

'I have briefly mentioned it over the phone and I don't think it sits right with my consultant at that moment in time – but since then there's been no discussions around it.'

[Exi208]

Further supporting quotes can be found in Table 3.

**Table 3:** Supporting quotes

*Expectations*	
Exi202	Why would I trade [what I have] for having to hike up to London every week or something for bloods for six months?
Exi203	I think if it’s a cure… It doesn’t cure all the joint replacements, it doesn’t cure the arthritis, it doesn’t cure the hepatitis, it doesn’t cure the cirrhosis—it just solves a problem, which is bleeding. That’s what it does, it solves a problem. It means that you don’t bleed anymore. Does that mean that haemophilia is cured? It may do. I just don’t… The word doesn’t sit right with me for some reason
Exi205	And then also, just the process itself. I thought at the time… I mean, when I was doing some reading, I was still at Uni and I thought no way I’m doing anything like this at Uni, going in and out of that process
	But just the process of going in and out of hospital multiple times… And then even when I went to [names hospital] last year and did some more reading about it at that stage, I thought it was going to get mentioned in some form of a conversation about what it is and the future, as it always does now. But I just thought I’m not so sure I want to make my immune system go to tatters when we’re part of a worldwide pandemic, and also, I’m not so sure this is the most responsible thing to be doing in an NHS Covid crisis, really
	There’s not enough evidence, not enough data behind that, and not enough people really going through the process of it yet
Exi208	At the moment, it’s a little bit, I feel, like… not a waste of time, but I just feel like if you’re going through it you’ll put a lot of strain probably on your family as well as your work. I just feel that’s probably quite a massive reason why I wouldn’t feel comfortable going along with it
	My lifestyle at the moment, it fits around my treatment process. So, I just kind of want to keep it as much as normal as I can
Exi211	They might be quite open to it, but I’ve only just started this job so… I’d have to… probably give it a while before I…
Exi212	Yes… I mean, thinking of where personally I am now with college and where I’ll be next year, if—if—I were to be, say, going on it next year, next year is one of the most important years for not only graduating college but then also setting up my professional life. So, that’s not something I’d really want to sabotage by going onto this, and then having the next X, Y, Z. The pay-off just wouldn’t be worth it when the medication that I’m on is allowing me to live the lifestyle that I currently am
Exi213	If we’re speaking exclusively on haemophilia, then I think it’s quite a gold mine because there’s just so many options out there. It’s better now to be growing up with haemophilia than it was ten years ago, and obviously ten years before that and so forth. Hopefully, children that are born with haemophilia A now, for example, they’re not going to have as much joint damage as I do
*Outcomes*	
Exi201	It’s an interesting question, because certainly when they talked about [grandson] having gene therapy, his mother had said a flat no until another few years until they see if there are any side-effects that come out
	Her reason is give it 20 years and see what happens. Because the last time we discussed it was in the very early days and she was saying we don’t know what the side-effects are, we don’t know what the long-term effects are, we don’t know how long the effects will last, we don’t know whether it will be worse after if they do come back
Exi202	Maybe in ten years when it is one and done, maybe that will be different
	If my treatment wasn’t performing as well, maybe that would be a different question, a different equation almost
Exi203	If they turned around and said, ‘Right, gene therapy is for you for all these reasons,’ and I ask, ‘Well, what are the chances of it working? What are the chances of it failing? What’s the chance of me never needing to inject again?’ You know, the fact that I’m 57… ‘What’s the risk of bleeds?’ To me, all the answers there are all unknowns because it’s gene therapy, it’s new
	I don’t think. I think, for me, it’s the way… I guess the way the language is all about curing haemophilia, and I find that a little bit uncomfortable in that haemophilia is therefore defined as this problem to be fixed, and therefore I am… there’s something wrong with me that needs to be corrected, rather than ‘Here’s some drugs that can allow you to live a flourishing life’
Exi205	For starters, everyone’s saying it could work, it might not work, so ok we’re in a 50/50 situation, flip of the coin anyway. And then it might work but you might only get to 12 percent. You might only get to 12% potentially, or you might be 95%. I mean, it really is a flip of the coin, and we don’t really know… And there’s no logical reason… there doesn’t seem to be a logical reason right now about where you sit
	I think there are so many other things that could come first to make haemophilia better at a far more reasonable cost and would have a far more direct impact on 99 percent of people if we started doing it tomorrow morning
Exi206	The same with gene therapy. There’s a lot of talk of roughly eight years because that’s as much data as we have, and maybe levels dwindle. So, especially getting older, would I take that one jab for eight decent years and then go back to being a normal haemophiliac? Maybe. It’s difficult to quantify it and to make the decision based on that because… it does work for a lot of people… it’s just you don’t know… It’s like a lottery, though—you go into it and you don’t know what level you’re going to get
	I think it’s the finality of gene therapy. Moving onto [Names FVIII Mimetic Product], if it doesn’t quite work for me—I know, and I’ve discussed it with my centre about switching to an extended half-life because that’s still an option. But if you go onto gene therapy and it doesn’t quite work, what situation are you left in?
Exi212	But then I also think… well, I also kind of like the security that I have with my medication, knowing that if I take my medication like I’m supposed to then nothing’s going to happen. Whereas with gene therapy I feel like there’s still a lot that’s kind of unknown
*Access*	
Exi202	It’s not been mentioned to me. Again, I have struggles trying to get them to elaborate on basic things like what’s happened to those scans you did, no I don’t want to be changed from the current treatment I’m on. So, no—no discussions about gene therapy
	I changed centre during Covid, so that’s been a huge barrier to just accessing a lot of things in general
Exi203	And I don’t know the politics behind the decision-making that different treatment centres have got. I don’t want to get involved in that, but I assume and I’m guessing that different treatment centres have got different views because of different levels of funding or whatever it might be
	But it is kind of ‘We think this is going to be good for you for these reasons,’ and I go, ‘Yes, if it stops me from bleeding then great, fantastic.’ I did push the [names treatment] one though. I did say, ‘I want to do this as soon as possible.’ So, that’s the only one time that I’ve gone in and said, ‘Can we stop faffing around with this once-every-two-days stuff? I want to change because I’m hearing great things.’ And they just said, ‘Yes, we’ll do it as soon you’re fit and ready.’ And we did and it’s been great
Exi204	So there is a financial part of it, but [my centre] is a big centre, similar to other areas, so I presume they have leverage in terms of how it would work… So, it’s just understanding how the decisions are made. But we’re not given a choice
	I think the NHS has to ultimately derive the greatest value from the smallest amount of money
Exi206	There are still some times where we butt heads, which… rightly so, because they’ll say one thing and I will try and argue my point against it. But it’s a much more open conversation. I’m not being dictated to as to what I need to do. They will listen to me and offer their advice, so it’s much better
	From everything I’ve heard about gene therapy, it costs a lot of money and it’s a bit of a 50/50 thing
Exi207	The centre I was at was not very helpful, would not refer me to the centre to get onto the trial—which at the time I was very annoyed about
Exi208	I don’t think I’d be one of the main patients to benefit from it in their eyes. So, when you look at the list of priorities—because it’s not going to go to everyone on that patient list—I wouldn’t be top of that list
Exi211	So, I didn’t even know it was a thing until I went on Facebook, to be honest. And then I spoke to a few more people around my age and they said they’re all on it, so I just wondered why I’ve not been offered it
Exi212	It was that they got a cheaper deal buying a bigger bunch of, say… I can’t remember what any of them are called, but they get a bigger deal for buying more of the same product, so they’ll buy that, put more patients out on it, because obviously it saves them more money at the end of the day. So, I understand that it’s not really a possibility to have “Oh, here’s all these different products for all these different patients who need what they need.” I understand the constraints on that
Exi213	At all the centres that I’ve been to it’s just not something that’s been brought up with me at all
*Information*	
Exi201	I learnt about it when my daughter told me about it. I’ve always ignored my haemophilia, I’m not part of any real haemophilia group or…I’m not a member of the Haemophilia Society and I don’t read things about it. And I don’t have any things that come into my inbox about it. It’s just one of those things I’ve tried to… well, I just have ignored. So, I heard about it when [she] told me about it
	Not that I’m aware of it. Mind you, I only have a five-minute conversation with my guy anyway, about every six months. He phones me up and says, “Are you doing all right?” and I say yes. He says, “Have you had any bleeds?” and I say no. And that’s it
Exi203	I think what has always frustrated me a little bit is there’s no sort of education about the different treatments in layman’s terms. I’m headlines and not detail, just like a lot of other people—I do the headline and the detail I expect layman’s terms information
Exi205	I think they should be putting more information out, but I’d like to know if they actually have the information themselves. That’s the thing I’m really curious about because I’m not so sure they do. I think that’s why they might all be… none of them are putting information out because I’m not sure they’ve potentially got this actual data to show us all yet
	And doing some reading about it… The thing I’ve found with reading about it is… like I said, being a person who’s focused by data, there just never was anywhere that told me the exact… not an exact number, but ranges were very coy and ranges were too big that I don’t want to get involved in that
Exi207	I don’t think so. I’d have to go search it and do a bit of a… like, you’d have to do a dive in the studies because it’s still in trial period, so the information’s not that easy to ask, like at the snap of your fingers
	So, my first consultation there took about three and a half hours because we literally sat there and went through everything. There are still some times where we butt heads, which… rightly so, because they’ll say one thing and I will try and argue my point against it. But it’s a much more open conversation
Exi208	But I think the thing for me is… I think I’d want to just see a bit more of the longevity results. And I think if I was to ever make the move… I think this time last year I was a definite no, but I think the idea around it is actually… I think it is going to probably be the future. So, for me, I’d probably actually want to speak to someone –when I say ‘someone’ I mean not my direct team but maybe someone who’s gone through the process, as such—first. But short term, the next 12 to 24 months, I probably would still be a no if it was offered
	Yes. I mean, I’m sure there is a vast amount of evidence and results out there. But the next step is probably accessing it and how easy it is to access it. I’d like to think I’m at a reasonable level, where I can go out and find that sort of stuff. But other than the AGM for The Haemophilia Society, I have found it quite hard to kind of find information, or up-to-date information anyway
Exi209	So, I think it’s a mixture of there is the info out there, but you’ve got to look for it, it’s not fed to you on a plate, and also hearing experiences from people who have actually been through it. I think if you’re not proactive to find someone like that then there’s not enough information
Exi211	I feel like there’s still not enough to go on here—which is fine, because it’s still new, they still don’t have as big a pool of people that are on it to say, “Here’s what our research shows”
	Getting the consultants to speak about it a bit more, really. [My consultant] doesn’t actually really tell me about anything that’s new. The nurse tries to but the consultant obviously leads the consultation, so…

## Discussion

All participants in this study saw gene therapy as a positive development in the treatment of haemophilia. However, they also expressed concerns that present barriers to gene therapy.

The most significant concern, related to the need for steroids following gene therapy and their potential side effects. Even though not all clinical trials necessitated the use of steroids [19, 20], there was a widespread perception that, following gene therapy, there would be a need to take of steroids for prolonged periods. The level of concern expressed about the side effects, particularly immunosuppression, may always be a general concern but may also have been heightened during the Covid-19 pandemic when the interviews were taking place. The concern may, therefore, decrease as the pandemic continues to wane. The prospect of further pandemics [21, 22] may, however, mean there will always be concern about immunosuppressive medication following gene therapy. Further research is needed to ascertain whether immunosuppression will remain a necessary feature of gene therapy or whether alternative strategies can be used.

In common with other studies [3, 23, 24], many participants had concerns about the outcomes of gene therapy. However, rather than focusing on factor expression and durability, these related more to the need for an increased level of engagement with clinical services in the first 6–12 months, and a perceived additional burden related to the treatment of bleeds.

The ability to treat bleeds quickly and effectively has been a key advantage of home treatment [25]. Participants appeared to be concerned that treating bleeds after receiving gene therapy would involve contact with or a need to attend their haemophilia centre, which represented a ‘backwards step’, even if bleed frequency was reduced.

Many participants also felt their current treatments did not significantly impact their lives and were, therefore, reluctant to consider a change that might increase the frequency of interaction with their treatment centre, even if it was only in the short term. Consequently, some said they would be hesitant to pursue gene therapy as a treatment option. Similar concerns were expressed in other Exigency sub-studies [13, 16]. This may highlight a genuine concern among PwH, but may also reflect another perceived barrier the participants discussed: a lack of accessible, patient-focused information.

A number of participants stated that the language used to discuss gene therapy was too complicated and not pitched at a level they could easily understand. As a result, some said they were unable to decide whether gene therapy would or would not be an appropriate treatment for them. There is, therefore, a need for gene therapy providers, haemophilia care teams and patient organisations to do more to enable all PwH to fully understand its nature and implications. This should include plain language summaries, patient education leaflets, visual materials and engagement events, with consideration given to individual communication needs [26].

Recent discussion about gene therapy, education and decision-making has focused on shared decision-making (SDM). The concept of SDM was first described in the late 1980s as a reaction against the paternalistic nature of decision-making [27, 28], but it was first used in the context of haemophilia care in 2014 [29]. SDM is based on a two-way exchange of information between patients and HCPs [30–32], and seeks mutual understanding and agreement between the medical knowledge of HCPs and the beliefs, preferences, and experiences of patients/caregivers [33–35]. There is a concern, however, that SDM may retain features of the paternalistic decision-making process it initially sought to resolve, including limiting the number of available options, framing and nudging [36–38], many of which seem to have been experienced by many participants in the study. Despite these concerns, the core concepts of SDM—active dialogue, mutual understanding and agreement—must remain central to the educational process to enable PwH to choose the most appropriate treatment option for them at any given time, whether gene therapy or another of the available therapies [39].

SDM to support access to haemophilia gene therapy, however, may be impacted by another concern raised by the participants: cost. Aware that treatment for haemophilia is expensive, and that their access to treatment has always been limited by its cost, many participants were anxious that haemophilia gene therapy may be yet more expensive. They were concerned that gene therapy might not be considered cost-effective and that, as a result, its availability would be limited or even prohibited, as has been seen in other conditions [40]. Indeed, at the time of writing, draft guidance from the National Institute for Health and Care Excellence (NICE) has not recommended the first gene therapy product as a treatment option in the UK [41]. It may be that until more long-term data regarding expression, durability, and efficacy is available, many PwH will remain unable to access gene therapy. Until such time that access is available to all, some commentators have suggested that stakeholders—patients, patient advocacy groups and clinicians—should seek to evidence the everyday realities and difficulties of living with haemophilia, including treatment burden and anxiety induced by the ongoing fear of potential bleeds [42].

### Strengths and limitations

Some aspects of this study may affect the generalisability of its findings. These relate to the size and structure of the study sample, the study setting, and the qualitative aspect of its methodology.

Participants in the study were under the care of centres not involved in gene therapy clinical trials. However, as this means they would have had less access to the information about gene therapy, the authors believe this makes them more representative of the majority of PwH in the UK. The sample included fewer people with haemophilia B than people with haemophilia A. This was not seen as a concern as the majority of the issues raised related to gene therapy processes, outcomes, education and access, and were not therefore, therapy or condition specific.

That the study was conducted in a high-income country where access to factor prophylaxis is readily available. Findings therefore may not be representative of the views of PwH in lower and middle-income countries. Further research should be undertaken in other economic territories to address this.

The nature of qualitative research makes it difficult to generalise any data collected. Its strength lies in the ability of qualitative inquiry to provide meaning and deeper understanding in specific situations [43, 44]. Through both meta-analyses and syntheses, qualitative sources can have application beyond their immediate context [45, 46]. This paper, therefore, should not be seen in isolation but within the growing body of qualitative haemophilia gene therapy studies [11, 13, 15, 47].

## Conclusion

This Exigency study subgroup sees gene therapy as a positive treatment development that promises a greatly improved QoL. However, four significant issues were identified that could present barriers to participants considering gene therapy as a treatment option: the processes involved, the outcomes of gene therapy, access to gene therapy, and the availability of information. If the promise of haemophilia gene therapy is to be realised, these barriers need to be acknowledged and addressed by HCPs, patient organisations, and gene therapy providers. If, however, it is not possible to resolve these barriers fully, it is essential that PwH are enabled, through a SDM process, to access an available treatment option that offers the QoL they seek.

## Material and methods

### Design

Exigency is a mixed methods study designed to explore the opinions of PwH and their families in the UK about gene therapy [17]. This sub-study focuses on PwH who have not yet had the opportunity to participate in a gene therapy programme because they are under the care of a centre not currently involved in ongoing clinical trials.

Following a brief indicative literature review, the study team and a patient representative designed a semi-structured interview schedule (See Additional file 1: Appendix 1). The guide addressed issues including the participant's condition, their treatment history, their understanding of gene therapy for haemophilia, why they think they have not yet been offered the possibility of taking part in a gene therapy study, and how gene therapy might be accessible in the future as a treatment option (18). The guide was used as a template for the interviews, but the interviewer (Principal Investigator) was free to explore any of the issues raised in more depth. He we also encouraged to explore any issue discussed in a previous interview if it had been discussed (unprompted) on at least two previous occasions.

### Recruitment

Participants were recruited through participant identification centre referral, social media advertising and word-of-mouth referral. The eligibility criteria allowed for the recruitment of all people with either haemophilia A or B who could give written informed consent and had a good command of English. All participants and a family member, if available, took part in a single one-hour semi-structured interview using a video conferencing platform. All interviews were carried out by the principal researcher (SF). Three of the 13 participants were known to the principal researcher prior to their participation in the study. Video conferencing was used during the Covid-19 pandemic as a social distancing strategy. It was continued post-pandemic as it was convenient and popular and has been shown to provide rich qualitative data [48–50].

### Data collection

#### Analysis

At consent, each participant was assigned an individual study number. All interviews were recorded (audio and video), transcribed, and thematically analysed inductively. NVivo for Mac (version 12) was used to facilitate the coding process. Field notes were made following each interview. All interviews were coded within five working days by the principal investigator. Seven of the interviews were randomly re-coded by a second investigator (KK) and both investigators met once a month to discuss all completed interviews to ensure the reliability of the interview process and code generation. Previously created field notes were also be reviewed to provide added context to the discussion. This process was continued until saturation was achieved and the interviews discontinued. Upon completion of the interviews, the study team reviewed all the emergent codes, refined them further, and identified the final themes (see Table 4).

**Table 4:** Coding diagram

Primary codes	Secondary codes	Themes
Changing goalposts	Concerns about the processes of gene therapy	Expectations
Immunosuppression		
COVID-19		
Increased burden		
Employment concerns		
Identity		
Increased medicalisation		
Reduced choices		
	Uncertainty about the results of gene therapy	Outcomes
Increased bureaucracy		
Cure		
Durability		
Benefits of		
Factor levels		
Not just gene therapy		
Anxiety		
	Access to treatment	Access
Infected blood		
Inhibitor		
Cost		
Gatekeeping		
Clinical trials		
Treatment centre		
	A lack of understanding of gene therapy	Information
Discussions about GT		
First Introduction to GT		
Understanding of GT		
Not enough information		
HCP don't understand it		
Unwillingness/Inability to discuss		
"I don't understand it"		
Not enough information		
Too complicated		

### Supplementary Information

**Additional file 1.** Exigency Interview guide.

## Data Availability

The datasets generated and/or analysed during the current study are not publicly available, as none of the participants consented to this. The data are available from the corresponding author on reasonable request.

## Declarations

### PPI Statement

The Exigency Protocol was written with the assistance of a patient representative (a person with haemophilia who has subsequently gone on to have gene therapy). This representative was also involved in the design of the interview schedule. The protocol was also reviewed before REC submission by two patient representatives: a woman with Haemophilia with an affected son, and a partner of a person with haemophilia.
