# Supplementary material for: Barriers to gene therapy, understanding the concerns people with haemophilia have: an exigency sub-study
Source: Orphanet J Rare Dis. 2024 Feb 10;19:59. doi: 10.1186/s13023-024-03068-2 (PMC10859013; doi:10.1186/s13023-024-03068-2)
Supplement: Supplementary file 1 — Additional file 1. Exigency Interview guide. [file 13023_2024_3068_MOESM1_ESM.docx]

##

## Interview guide – PWH who did not undergo gene therapy

## Preamble

- Thank you for agreeing to take part in this project about how people feel about having undergone or considered undergoing gene therapy. Everything you tell us in this interview will be treated with complete confidence – your identity will never be revealed.

***I’d like to start by asking a few questions about you and your haemophilia:***

- Can you begin by telling me how old you are and a little bit about what hobbies you have?
- Can you tell me about your haemophilia – when were you diagnosed?
- What was it like for you growing up with haemophilia?
- Did you lose much time from school?
- What treatments have you been on in the past, and what treatment are you on now?
- What is the worst thing about your treatment?
- Can you recall how many bleeds you had in an average year?
- How are your joints?
- Did you have any joints that bleed more than others?
- How did you manage these bleeds?
- Do you have any mobility issues?
- Have you ever had an operation in hospital? What was it for?
- Have you ever done any haemophilia trials before?
- Why did you decide to take part?
- Who suggested taking part?
- How did you find being part of that study?
- Did you find any part of it difficult?
- ***Now I’d like to talk about your understanding of gene therapy***
- When and how did you first hear about gene therapy?
- What do you know about gene therapy for haemophilia?
- Do you feel that you know enough about gene therapy?
- Do you have any concerns about gene therapy?
- Have you known anyone who has had gene therapy?
- Have you discussed gene therapy with your doctors?
- What have they said about it?
- Have you spoken to your family about gene therapy?
- What do they think about it?
- Is gene therapy something that you would consider having?
- If yes, why?
- If not, why?
- ***Now I would like to go on to ask you about the future****:*
- What are your goals?
- In the next six months
- In the next five years?
- What are your hopes/expectations for the treatment of people with haemophilia?
- Have you heard about any other treatments that might be available in the future?
- Do any of these treatments look more or less attractive than your current treatment or gene therapy?
- What advice would you give to others considering gene therapy?

## Closing

- Is there anything else you would like to say or ask of me?
- I would once again like to thank you for your time today. Once we have completed all the interviews we aim to publish all the results but will of course maintain the anonymity of you and anyone else who took part in the study. Once the paper is published, we will send you a copy if you wish.
- Goodbye
